# Single-incision laparoscopic enucleation for pancreatic insulinoma with preoperative nasopancreatic stent placement: A case report

**DOI:** 10.1016/j.ijscr.2022.107115

**Published:** 2022-04-21

**Authors:** Hiroyuki Tsukayama, Takeyuki Misawa, Makoto Watanabe, Hideki Takahashi, Takashi Koenuma, Rie Kondo, Hiroe Toyoda, Makoto Shibuya, Keita Wada, Keiji Sano

**Affiliations:** Department of Surgery, Teikyo University School of Medicine, 2-11-1 Kaga, Itabashi-Ku, Tokyo 173-8606, Japan

**Keywords:** SILE, Single incision laparoscopic enucleation, LE, Laparoscopic enucleation, POPF, Postoperative pancreatic fistula, MPD, Main pancreatic duct, NPS, Nasopancreatic stent, CT, Computed tomography, EUS, Endoscopic ultrasonography, SACI test, Selective arterial calcium injection test, IOUS, Intraoperative ultrasonography, LCSs, Laparoscopic coagulating shears, EPS, Endoscopic pancreatic stent, SILS, Single incision laparoscopic surgery, SIL-DP, Single incision laparoscopic distal pancreatectomy, Insulinoma, Single-incision laparoscopic surgery, Enucleation, Nasopancreatic stent

## Abstract

**Introduction and importance:**

Most insulinomas are benign and solitary, with a tumor diameter less than 2 cm; therefore, laparoscopic enucleation, which is a minimally invasive procedure that can preserve the pancreatic parenchyma, is considered an optimal procedure. The key to enucleation is to avoid injury to the main pancreatic duct (MPD). Herein, we present a case in which single-incision laparoscopic enucleation (SILE) was performed for insulinomas, with preoperative nasopancreatic stent (NPS) placement.

**Case presentation:**

A male patient in his fifties underwent SILE for insulinomas. To prevent injury to the MPD, an NPS was preoperatively placed. All surgical procedures were performed through a single mini-laparotomy site in the umbilicus. NPS placement facilitated identification of the MPD under laparoscopic ultrasonography. Enucleation was successfully completed without any injury to the MPD, and the NPS was removed immediately after confirming that there was no injury to the MPD by the NPS via pancreatography. The postoperative course was uneventful.

**Clinical discussion:**

This report serves to highlight the maximum safety and minimal invasiveness of SILE with the preoperative NPS placement. Preoperative NPS placement is useful for avoiding injury to the MPD during enucleation and has the merit of helping to recognize whether leakage occurs by intraoperative pancreatography via the NPS.

**Conclusion:**

Preoperative NPS placement helps to ensure the safe enucleation of pancreatic insulinomas even in single-incision laparoscopic surgery, with minimal invasiveness and better cosmetic outcomes.

## Highlights

• Single-incision laparoscopic enucleation (SILE) is a challenging procedure.
• SILE is minimally invasive and offers better cosmetic outcomes.
• To avoid injury to the main pancreatic duct, a nasopancreatic stent (NPS) was placed.
• SILE can be performed safely via preoperative NPS placement.

## 1 Introduction

Insulinoma is a rare functional neuroendocrine tumor of the pancreas [1]. Most cases are benign solitary tumors with diameters less than 2 cm [1]. Pancreatic enucleation is commonly performed for insulinoma, with the goal of achieving both radical resection and preservation of the pancreatic parenchyma [2]. Furthermore, laparoscopic enucleation (LE) should be considered an optimal procedure because of its minimal invasiveness [3], [4].

The most significant complication of LE is postoperative pancreatic fistula (POPF), mainly due to injury to the main pancreatic duct, resulting in increased morbidity. In several reports, the frequency of POPF after pancreatic enucleation is still high, ranging from 9% to 69% [5].

The key to safely performing enucleation is avoiding injury to the main pancreatic duct (MPD). Herein, to prevent injury to the MPD and to minimize the invasiveness of laparoscopic enucleation, we performed single-incision laparoscopic enucleation for an insulinoma with preoperative placement of a nasopancreatic stent (NPS). This case report has been reported in line with the SCARE criteria [6].

## 2 Presentation of case

**Fig. 1:**
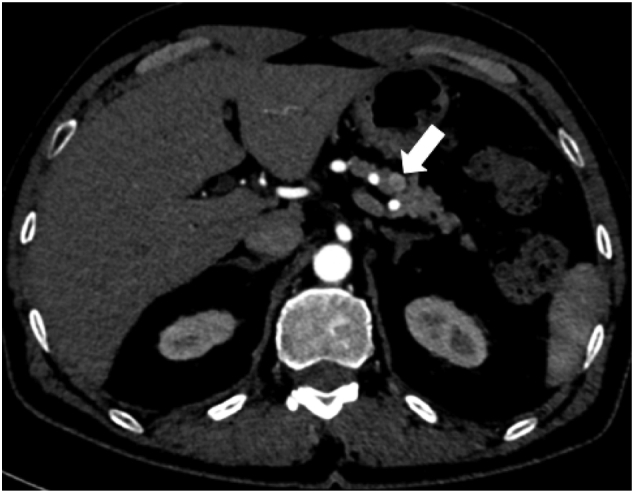
Preoperative contrast-enhanced computed tomography. A hypervascular tumor that was 10 mm in diameter in the pancreatic body was revealed.

**Fig. 2:**
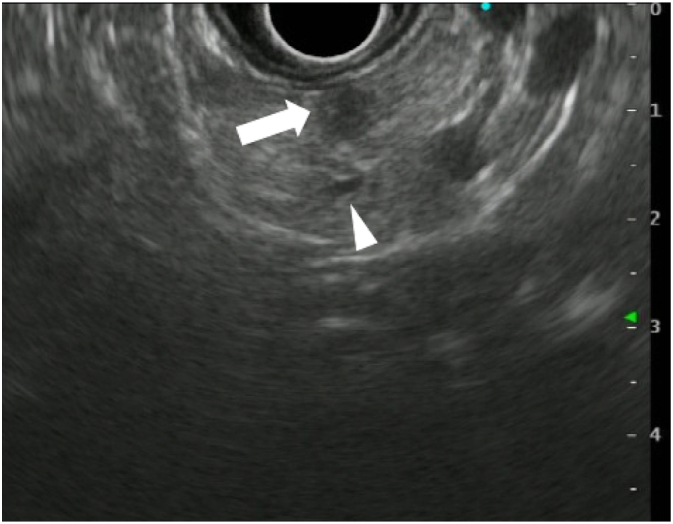
Preoperative endoscopic ultrasonography revealed that the distance between the tumor (arrow) and the main pancreatic duct (arrowhead) was approximately 3 mm.

A male patient in his fifties was presented to the emergency department with loss of consciousness due to hypoglycemia. His past medical history was significant for several times of loss of consciousness without a known cause, and he had no history of any abdominal surgeries. Contrast-enhanced computed tomography (CT) revealed a hypervascular tumor 10 mm in diameter in the pancreatic body (Fig. 1). Endoscopic ultrasonography (EUS) showed a hypoechoic tumor with a well-defined margin, in the pancreatic body. The distance between the tumor and the main pancreatic duct (MPD) was 3 mm on EUS (Fig. 2). Insulinoma was diagnosed by a selective arterial calcium injection test (SACI test). A 5 Fr NPS (Cook Medical) was placed the day before the operation to detect injury to the MPD during the operation.

**Fig. 3:**
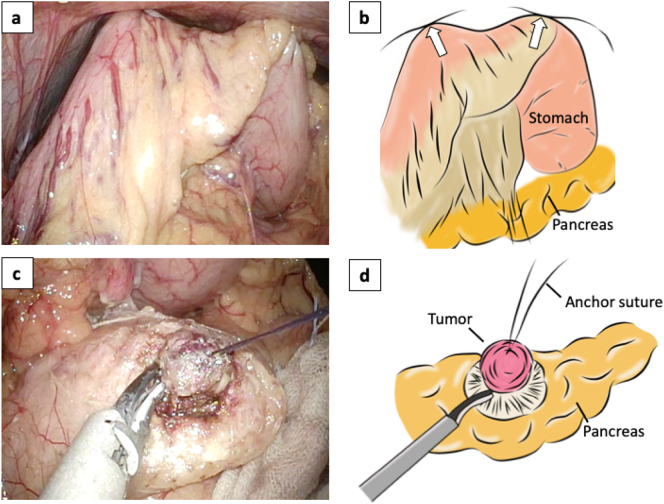
Intraoperative findings. The stomach wall was lifted with two 3–0 sutures (arrows) to achieve a better operative field of view (a, b). The tumor was lifted by 3–0 anchor sutures to visualize the resection line and then resected using laparoscopic coagulating shears (c, d).

**Fig. 4:**
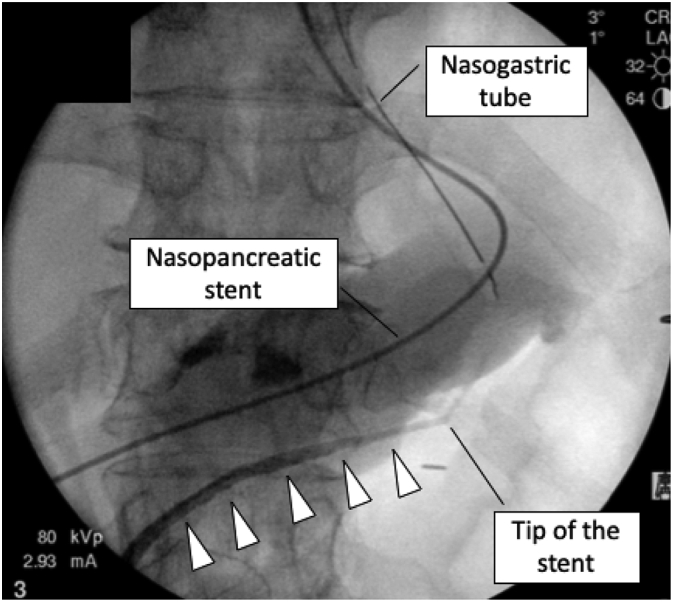
Intraoperative pancreatography immediately after tumor enucleation revealed no leakage from the main pancreatic duct (arrowhead).

All surgical procedures were performed through an X gate® (Sumitomo Bakelite Co., LTD), which was placed in a single mini-laparotomy site in the umbilicus by the hepatobiliary surgery team. Pneumoperitoneum pressure was automatically maintained at 10 mmHg by a CO2 insufflator. First, after the omental bursa was opened, the great curvature of the stomach was lifted to the abdominal wall with two 3-0 sutures to obtain a better operative field of view (Fig. 3a, b). The tumor, which was slightly reddened and protruded on the surface of the pancreatic body, was visualized. The relationship between the tumor and the MPD was well recognized by intraoperative ultrasonography (IOUS) owing to hyperechoic images of NPS. IOUS provided a clearer image than preoperative EUS and revealed that the tumor was away from the splenic vessels, and the distance between the tumor and the MPD was 2.5 mm. Enucleation was performed as scheduled. The pancreatic parenchyma was resected along the surface of the tumor using laparoscopic coagulating shears (LCSs). The tumor was lifted by 3–0 anchor sutures to visualize the resection line (Fig. 3c, d), and IOUS was used from time to time to confirm that the resection line was correct and that there was a suitable distance from the MPD. Both arterial and venous bleeding were controllable with gauze packing and soft coagulation. Finally, a relatively thick feeding artery was identified at the bottom of the tumor and was reliably sealed and dissected. After extirpation of the specimen, intraoperative pancreatography via NPS was carried out using meglumine diatrizoate (urografin) to determine whether injury to the MPD had occurred. No apparent leakage was found, and NPS was removed immediately after pancreatography (Fig. 4). Fibrin glue and a tissue sealing sheet (TachoSil® CSL Behring) were applied to the enucleated site, and then a closed drain was placed close to the enucleated site.

The bleeding volume was 30 ml, and the operative duration was 329 min. The drain was removed on postoperative day 6, the patient was discharged without any adverse events on postoperative day 10, and the surgical wound was almost invisible. The pathological diagnosis was grade 1 pancreatic neuroendocrine tumor (G1) consistent with insulinoma. The patient is doing well with no tumor recurrence or onset of diabetes one year after surgery.

## 3 Discussion

This report describes the efficacy and safety of enucleation for insulinoma with the preoperative placement of NPS; single-incision laparoscopic enucleation should be considered an optimal procedure with minimal invasiveness.

Enucleation for benign pancreatic tumors is the most appropriate procedure to preserve the function of the pancreas, as it provides a better long-term function of the pancreas [7]. When enucleation for these tumors is selected, preoperatively confirming the relationship between the tumor and the MPD is essential to prevent pancreatic duct injury, which may cause severe POPF, resulting in catastrophic outcomes. However, more patients suffer from clinically relevant pancreatic fistula after tumor enucleation with either laparotomy or laparoscopic approaches [2], [7]. Fernandez-Cruz et al. described that overall postoperative complications, which are mainly pancreatic fistulas, were significantly higher in laparoscopic enucleation than in other procedures, such as laparoscopic distal pancreatectomy [8].

Several reports have described preoperative placement of pancreatic duct stenting for the prophylaxis of pancreatic fistula due to MPD injury during enucleation or partial resection [5], [9], [10], [11], [12], [13], [14]. In these reports, the strategy using mainly endoscopic pancreatic stents (EPSs) has been reported to have some advantages. First, with pancreatic duct stenting, the MPD can be well recognized by IOUS, allowing the relationship between the tumor and the MPD to be confirmed and the proper resection line to be secured [9], [14]. Second, it allows a major injury to the MPD to be visually detected by the stent color.

Additionally, NPS allows a minor injury to be detected by intraoperative pancreatography.

Conversely, preoperative pancreatic duct stenting placement does increase the risk of pancreatitis. Okamoto et al. described that, regarding the timing of preoperative stenting, a stent should be placed at a time point as close to surgery as possible because pancreatitis, if it occurs, makes pancreatic surgeries more difficult [10]. In our case, the NPS was placed the day before surgery, and despite a slightly elevated serum amylase level, there seemed to be no influence on the surgery. Although the optimal timing of removing the NPS is not unclear, early removal should be considered because it may cause postoperative pancreatitis.

However, if pancreatic disruption is suspected, longer-term placement is recommended because it could be one of the treatments for pancreatic fistula [12].

Laparoscopic pancreatic surgery is now widely performed, and several advantages have been described, such as reducing postoperative pain, accelerating recovery, and decreasing the incidence of wound-related complications as well as morbidity [15]. Furthermore, a great deal of interest in enhancing cosmetic outcomes and minimizing invasiveness have led single incision laparoscopic surgery (SILS) to be widely performed in many fields of abdominal surgery [16], [17]. Since Barbaros U et al. first reported single-incision laparoscopic distal pancreatectomy (SIL-DP) with splenectomy in 2010 and Misawa et al. reported spleen-preserving SIL-DP in 2012, several more reports of SIL-DP with or without splenectomy have been published [15], [18], [19], [20]. Yao et al. reported that for experienced laparoscopic surgeons, SIL-DP is feasible and safe and yields excellent cosmetic effects [18].

Compared with SIL-DP, single-incision LE for pancreatic tumors might be a less complicated technique because division of large vessels such as the splenic artery and splenic vein is not needed. Although it might take longer to perform SILE than conventional LE, enucleation might be suitable for SILS because it requires less movement of laparoscopic instruments due to the narrow and limited operative field. Misawa et al. suggested that the greater curvature of the stomach should be suspended with two stay sutures to the abdominal wall to obtain a better operative field for exposure of the pancreatic surface [15]. Using the same technique, in our case, we could successfully and safely complete enucleation without injury to the MPD thanks to preoperative NPS placement, and could provide a high degree of satisfaction in terms of cosmetic outcomes.

## 4 Conclusion

Preoperative placement of NPS has clinical value for the safe enucleation of pancreatic insulinoma even in single-incision laparoscopic surgery, with minimal invasiveness and better cosmesis.

## Provenance and peer review

Not commissioned, externally peer-reviewed.

## Sources of funding

None.

## Ethical approval

In our institute, the approval of the ethics committee for the retrospective analysis of a clinical case report is not required.

## Consent

Written informed consent was obtained from the patient for publication of this case report and accompanying images. A copy of the written consent is available for review by the Editor-in-Chief of this journal on request.

## Research registration number

Not applicable.

## CRediT authorship contribution statement

All authors contributed to the study. HT wrote the manuscript and drew figures. KW, TM, and HT performed the surgery. All authors read and approved the final manuscript.

## Declaration of competing interest

The authors have no competing interests.
